# The Effects of *N*-Acetylcysteine on the Rat Mesocorticolimbic Pathway: Role of mGluR5 Receptors and Interaction with Ethanol

**DOI:** 10.3390/ph14060593

**Published:** 2021-06-20

**Authors:** Sandra Fernández-Rodríguez, Claudia Esposito-Zapero, Teodoro Zornoza, Ana Polache, Luis Granero, María José Cano-Cebrián

**Affiliations:** Department of Pharmacy and Pharmaceutical Technology and Parasitology, University of Valencia, 46010 València, Spain; Sandra.Fernandez-Rodriguez@uv.es (S.F.-R.); clauesza@alumni.uv.es (C.E.-Z.); teodoro.zornoza@uv.es (T.Z.); ana.polache@uv.es (A.P.)

**Keywords:** *N*-acetylcysteine, alcoholism, glutamate

## Abstract

*N*-acetylcysteine (NAC) is a prodrug that is marketed as a mucolytic agent and used for the treatment of acetaminophen overdose. Over the last few decades, evidence has been gathered that suggests the potential use of NAC as a new pharmacotherapy for alcohol use disorder (AUD), although its mechanism of action is already being debated. In this paper, we set out to assess both the potential involvement of the glutamate metabotropic receptors (mGluR) in the possible dual effect of NAC administered at two different doses and NAC’s effect on ethanol-induced activation. To this aim, 30 or 120 mg/kg of NAC was intraperitoneally administered to rats with the presence or absence of the negative allosteric modulator of mGluR5 (MTEP 0.1 mg/kg). Thereafter, the cFOS IR-cell expression was analyzed. Secondly, we explored the effect of 120 mg/kg of NAC on the neurochemical and behavioral activation induced by intra-VTA ethanol administration (150 nmol). Our results showed that the high NAC dose stimulated cFOS expression in the NAcc, and that this effect was suppressed in the presence of MTEP, thus suggesting the implication of mGluR5. Additionally, high doses could attenuate the ethanol-induced increase in cFOS-expression in the NAcc, probably due to a phenomenon based on the long-term depression of the MSNs. Additional experiments are required to corroborate our hypothesis.

## 1. Introduction

*N*-Acetylcysteine (NAC) is a prodrug of the natural amino acid, cysteine, which has a long-established safety record among adults and children [1]. This drug is available as a mucolytic agent [2], and it is also used as an oral or intravenous antidote to treat acetaminophen overdose [3,4]. NAC is also a cystine prodrug. As such, several studies carried out using rats have demonstrated that NAC is able to increase the extrasynaptic glutamate (Glu) levels in critical brain regions, such as the nucleus accumbens (NAcc) core, through the glial cystine/glutamate exchange system (xC^−^), which exchanges extracellular cystine for intracellular Glu in an equimolecular mode [5,6].

Nonsynaptic Glu can interact with metabotropic Glu receptors (mGluRs). Group I mGluRs (mGluR1 and mGluR5) are positively linked to phospholipase C. Therefore, the direct activation of mGluR1/5 receptors results in increased phosphoinositide turnover, whereas Group II mGluRs (e.g., mGluR2 and mGluR3, among others) negatively modulate excitatory Glu transmission at the perisynaptic and postsynaptic levels through the inhibition of adenylyl cyclase [7]. Group I mGluRs are located away from synaptic zones and appear to be restricted to postsynaptic terminals [8]; Group II receptors are located both in glial cells (mGluR3) [9] and presynaptically, also away from synaptic zones (mGluR2) [10]. It has been suggested that Glu has a higher affinity for mGluR2/3 than for other metabotropic receptors, such as mGluR5 [7]. Therefore, low extracellular Glu levels should preferentially activate mGluR2/3 and attenuate Glu release from the active presynaptic terminals. However, higher levels of Glu should be able to activate not only presynaptic mGluR2/3 but also the low-affinity postsynaptic mGluR5s. In the present paper, we set out to analyze whether NAC is able to interact with mGluR receptors in a dose-dependent manner. If the effects of NAC on the extracellular Glu levels are dose-dependent, we should expect that low doses of NAC may produce a preferent activation of presynaptic mGluR2/3. On the other hand, high doses should produce the activation of both mGluR2/3 and mGluR5. These dual effects of low and high doses of NAC were observed by Kupchik and colleagues in 2012 using electrophysiological recordings in rat brain slices. Moreover, the behavioral consequences of these dose-dependent changes in extracellular Glu levels in the NAcc core were also explored [11].

Previous studies had already analyzed the consequences of the activation of mGluR2/3 on drug-seeking behaviors so that the agonists of these receptors, such as LY379268, attenuated some forms of drug-seeking behavior [12,13] and modified several neurobehavioral consequences of acute and chronic psychostimulant or opiate administration [14]. Zhao et al. (2006) showed that LY379268 can attenuate both stress and cue-induced, ethanol-seeking behavior, and it is additionally able to modulate cFOS early gene expression in the hippocampus and amygdala [15]. Recent experiments carried out in our laboratory have also used the alcohol deprivation effect (ADE) animal model to demonstrate the ability of NAC to prevent alcohol relapse, although the involvement of mGluR2/3 was not explored [16]. Other authors have recently reported the ability of NAC, administered intraperitoneally (i.p.) at a dose of 120 mg/kg, to block the development of ethanol-conditioned place preference without altering dopaminergic transmission [17]. Therefore, although the involvement of mGluR2/3 in the prevention of alcohol-seeking behaviors seems plausible, whether the dose-dependent NAC modulatory effect on synaptic Glu transmission is relevant in the prevention of alcohol relapse in animal models of alcoholism remains to be confirmed.

In the present paper, we initiated the exploration of this hypothesis through two experiments. In the first experiment, we analyzed the activating effect of high and low systemic doses of NAC on the synaptic Glu transmission onto the NAcc neurons by evaluating the increase in the cFOS expression in this brain area in rats, as well as the influence of 3-[(2-methyl-1,3-thiazol-4-yl) ethynyl]-pyridine (MTEP), a selective negative allosteric modulator of mGluR5, in preventing the cFOS expression increase. In the second experiment, we explored the effects of the administration of a high NAC dose (120 mg/kg) on the neurochemical and behavioral activation of the mesocorticolimbic pathway, which was induced via the intra-ventral tegmental area (VTA) administration of ethanol [18,19]. In this study, we have used cFOS IR-cell expression as a tool to measure the activation of the mesocorticolimbic system, according to the studies provided by other authors [20]. In addition, this technique allows for the determination of the activation of the VTA in the main projection area (i.e., the NAcc) as well as the measurement of the increase in IR-cell expression as a metabolic consequence of postsynaptic activity [21].

## 2. Results and Discussion

### 2.1. Experiment 1: Effect of MTEP on the Blockade of cFOS IR-Cell Expression in the NAcc after i.p. Administration of NAC

**Figure 1:**
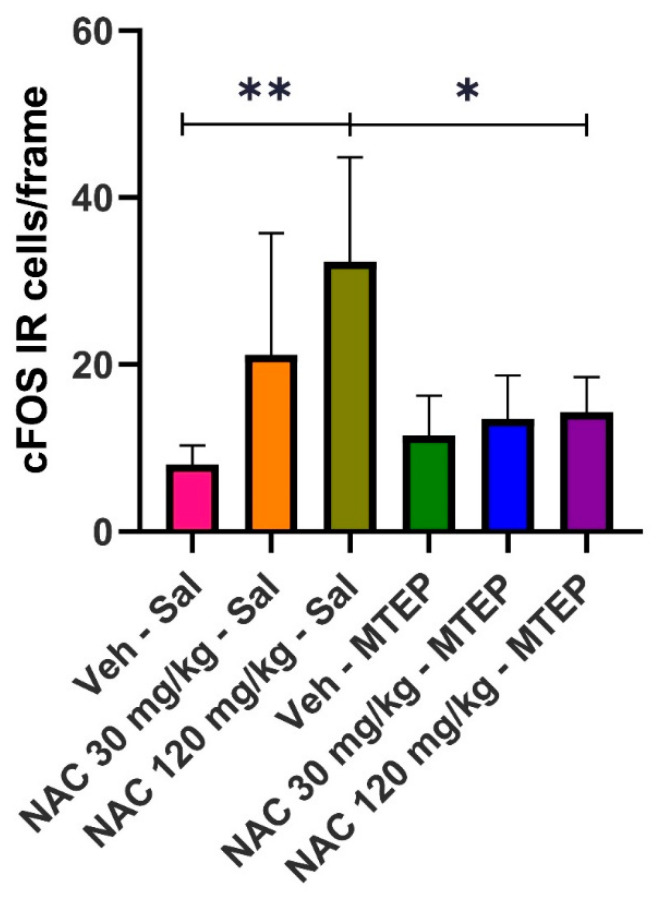
Effect of MTEP administration on cFOS IR-cell expression, induced by low (30 mg/kg) and high doses (120 mg/kg) of NAC. Data are mean ± SD, and represent the number of cells per frame. Asterisks indicate the existence of statistical differences between the marked groups. (Tukey’s test: * *p* < 0.05, ** *p* < 0.01).

**Figure 2:**
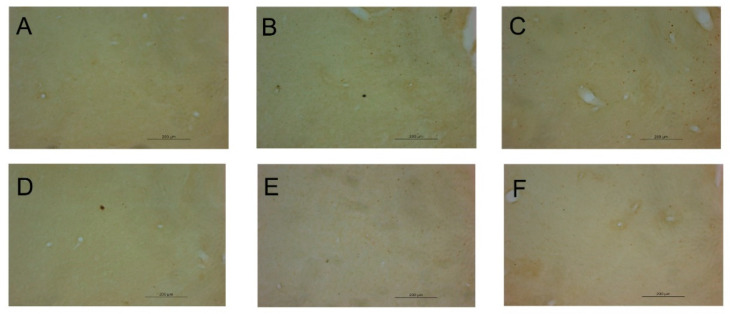
Representative images of coronal sections of the NAcc after cFOS staining in experiment 1. Legend: (**A**) group Veh/Saline; (**B**) group NAC 30/Saline; (**C**) group NAC 120/Saline; (**D**) group Veh/MTEP; (**E**) group NAC 30/MTEP; (**F**) Group NAC 120/MTEP.

Figure 1 shows that i.p. injection of 30 mg/kg of NAC (low dose) slightly increases the expression of IR cFOS cells in NAcc, while a higher dose (120 mg/kg) clearly increases their expression compared to the control group (Veh/Sal). The simultaneous administration of MTEP (0.1 mg/kg) apparently blocks the increase in IR cFOS cell expression in the NAcc, which is induced through the administration of the higher NAC dose; however, there is no relevant effect at the lower dose. Representative images of the coronal sections after cFOS staining in the NAcc are available in Figure 2.

Statistical analysis confirmed the above observations. A one-way ANOVA revealed the existence of statistically significant differences among groups (F(5, 27) = 5.807, *p* = 0.0009). A post hoc analysis (Tukey’s test) showed that the results obtained in the Veh/Sal group significantly differed with respect to the NAC 120/saline group (*p* = 0.0019). Moreover, the results obtained in the NAC 120/saline group significantly differed from those obtained in the NAC 120/MTEP group (*p* = 0.0127), suggesting the efficacy of MTEP in blocking the expression of cFOS IR-cells triggered by the high dose of NAC assayed. The 30 mg/kg dose of NAC did not significantly elevate cFOS IR-cell expression. However, it is evident that, although the increase in cFOS was not statistically significant, there was a slight trend to increase the immunoreactivity of cFOS. It is probable that a much lower dose of NAC than the 30 mg/kg dose employed in our study should have been administered to avoid an interaction with the mGlur2.

As described by several authors, the pharmacological actions of NAC include the restoration of the glutamatergic function in substance use disorders [2,22]. In this sense, NAC is able to interact with Glu transporters so that the indirect activation of the antiporter xC^−^ system, which is mainly expressed on astrocytes in cerebral grey matter, can increase Glu levels in the extrasynaptic space in the NAcc core [11,23,24]. On the other hand, several neurochemical studies have shown that the electrical or chemical stimulation of glutamatergic areas with afferences in the NAcc is accompanied by a significant and persistent induction of cFOS early gene expression. Several authors have reported an increase in cFOS expression in the NAcc after the chemical stimulation of glutamatergic areas, such as the ventral hippocampus. Along this line, Bargett and Henry detected that the expression of the protein product of the immediate early gene (cFOS) was increased in the NAcc shell after ventral hippocampal N-methyl-d-aspartate (NMDA) infusion in rats [25]. Other experiments carried out in our laboratory have reported the same effect after retrodialysis NMDA infusion into the hippocampal subiculum [20]. It could be suggested that the i.p. administration of a high dose of NAC would provoke an important increase—especially in extrasynaptic Glu levels—that would lead to an activation of the NAcc medium spiny neurons (MSNs) and would consequently increase cFOS IR-cell expression in this area.

Our results from using MTEP are in agreement with those reported by Kupchik et al. and seem to confirm the dual role displayed by extracellular Glu in NAcc activation. In their electrophysiological studies on rat brain slices, the authors showed that 5 µmol/L of MTEP did not alter the decreased excitatory postsynaptic currents (EPSCs) amplitude evoked by 0.5 µmol/L of NAC (low dose), whereas the same concentration of MTEP was able to counteract the increase of 500 µmol/L of NAC-(high dose) induced EPSCs amplitude [11]. These authors concluded that the excitatory effects of a high NAC dose were mediated by post-synaptic mGluR5. Although we used a different experimental procedure, our results are in the same line, since 0.1 mg/kg of MTEP was able to block the increase in cFOS IR-cell expression evoked by a high dose of NAC. Moreover, the low NAC dose had no effect on cFOS IR-cell expression in the NAcc. Our results seem to indicate that the effect of a high dose of NAC could be mediated by the postsynaptic mGluR5 receptor. As these genes appear to be induced as a metabolic consequence of post-synaptic activity [21], it could be plausible that the blockade of the post-synaptic receptors (mGluR5) may lead to the abolition of the cFOS IR-cells’ expression. Further experiments involving other antagonists of mGluRs type I and II are necessary to corroborate our hypothesis.

### 2.2. Experiment 2: Effect of 120 mg/kg of NAC on Ethanol-Induced Activation of the Mesocorticolimbic System

**Figure 3:**
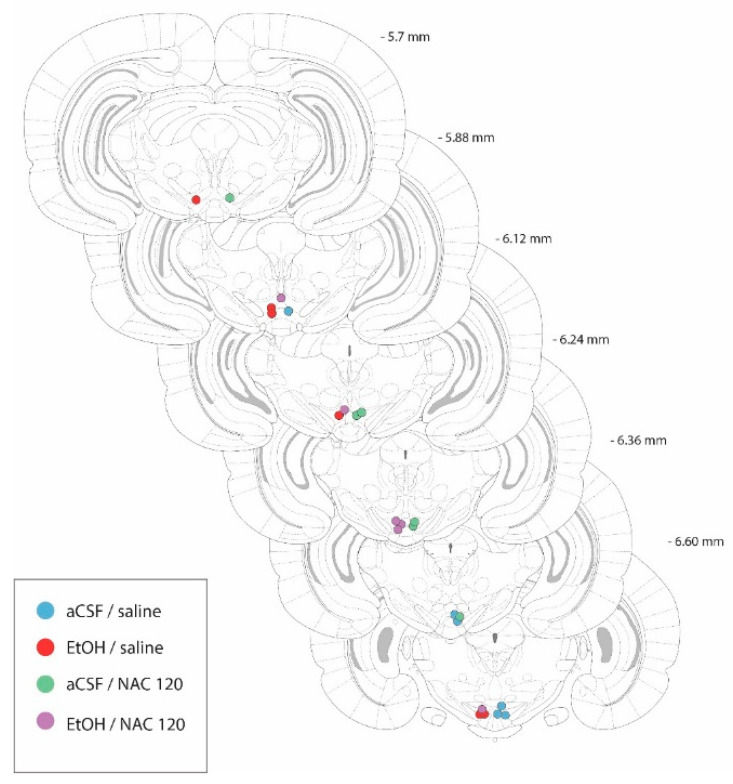
Diagram of coronal sections from the brains of rats used in experiment 2, indicating the placement of the tip of the injection cannulae in the posterior VTA. In order to facilitate the inspection of the figure, the placements from animals belonging to the aCSF-treated groups are shown on the right-hand side of the sections, whereas the placements from the EtOH-treated animals are shown on the left-hand side. Numbers indicate the distance from the anterior coordinate to the bregma. Adapted from Paxinos and Watson [26].

The cannulae placements after the histological evaluation of the rat brains used in this experiment are shown in Figure 3. All animals included in experiment 2 showed a correct position of the cannula tip in the posterior VTA (between −6.72 mm and −5.64 mm from bregma, Figure 2), according to Sanchez-Catalán et al. [19].

#### 2.2.1. Effect of NAC on the Increase in Locomotor Activity Caused by Intra pVTA Administration of 150 nmol of Ethanol

**Figure 4:**
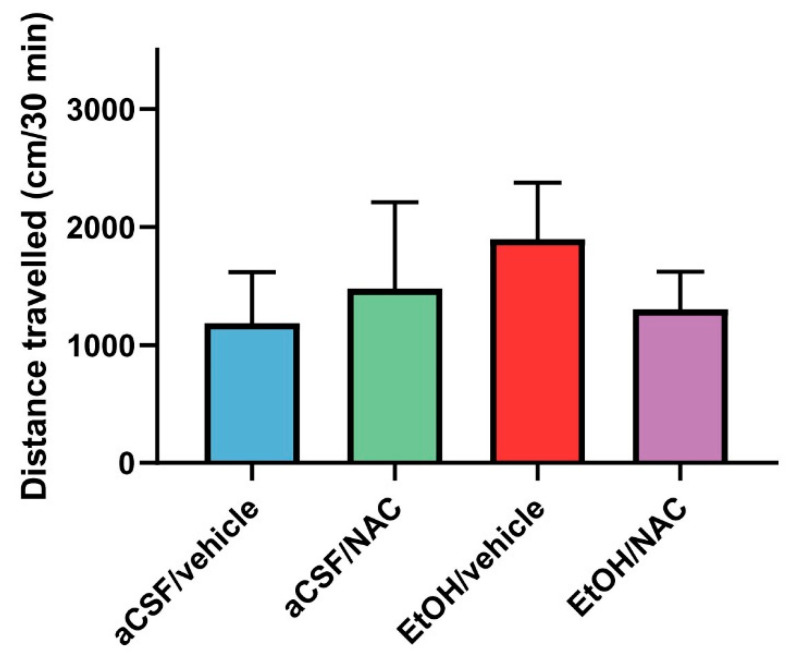
The effects of NAC administration (120 mg/kg), 30 min prior to the intra-VTA administration of EtOH (150 nmol), on the locomotor activity of rats. Data are mean ± SD and represent the distance traveled in 30 min. The Kruskal–Wallis test revealed no statistical differences between the groups.

Figure 4 represents the mean values of the distance traveled over 30 min that were obtained for each experimental group in this experiment. As can be seen in the figure—after intra-VTA EtOH administration—a clear trend toward the increase in the locomotor activity of animals, with respect to the aCSF/vehicle group (records increased from 1183 ± 430 to 1897 ± 480 cm), was observed. In addition, i.p. pre-treatment with 120 mg/kg of NAC also showed a trend to attenuate the above-mentioned increase in the locomotor activity induced by ethanol. However, the non-parametric Kruskal–Wallis test did not detect statistically significant differences (*p* = 0.1115).

The results of experiment 2a may suggest that the infusion of EtOH directly into the posterior VTA results in the induction of locomotor activity in Wistar rats, although this induction was not statistically significant. The activation of locomotor activity after the intra-VTA administration of 150 nmol of EtOH has already been validated several times in previous experiments that have been carried out in our laboratory using the same experimental procedures [18,19]. However, although in the present experiment an increase in the distance traveled is shown, there are no statistical differences between the groups. From a critical point of view, we suggest that the lack of statistical differences is probably due to the limited number of animals included in the present study.

#### 2.2.2. Effect of NAC on the Blockade of cFOS IR-Cell Expression in the NAcc Caused by Intra-pVTA Administration of 150 nmol of Ethanol

**Figure 5:**
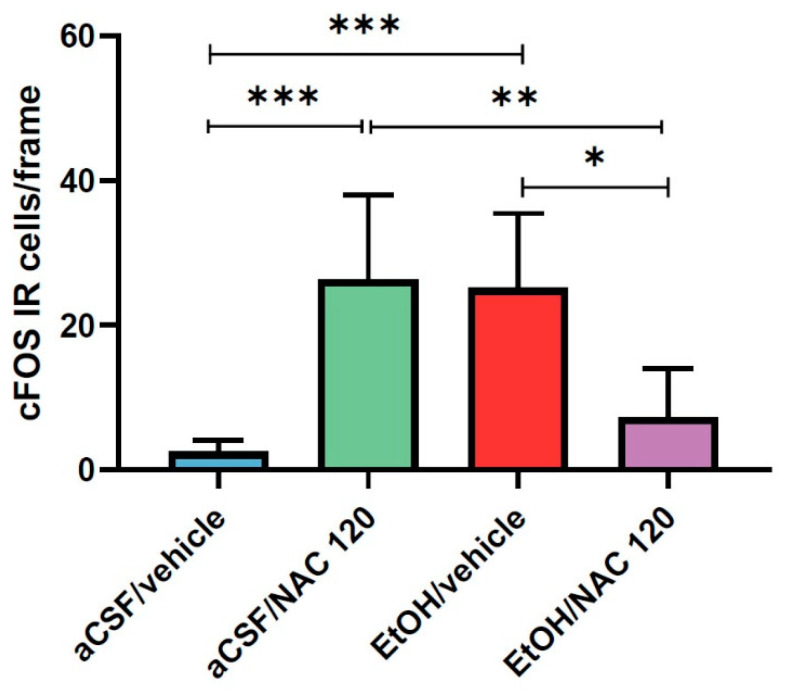
Effect of NAC administration (120 mg/kg) on cFOS IR-cell expression in the NAcc, 30 min prior to the intra-VTA administration of EtOH (150 nmol). Data are mean ± SD and represent the number of IR-cells per frame. Asterisks indicate statistical differences between the marked groups. (Tukey’s test: * *p* < 0.05, ** *p* < 0.01, *** *p* < 0.001).

**Figure 6:**
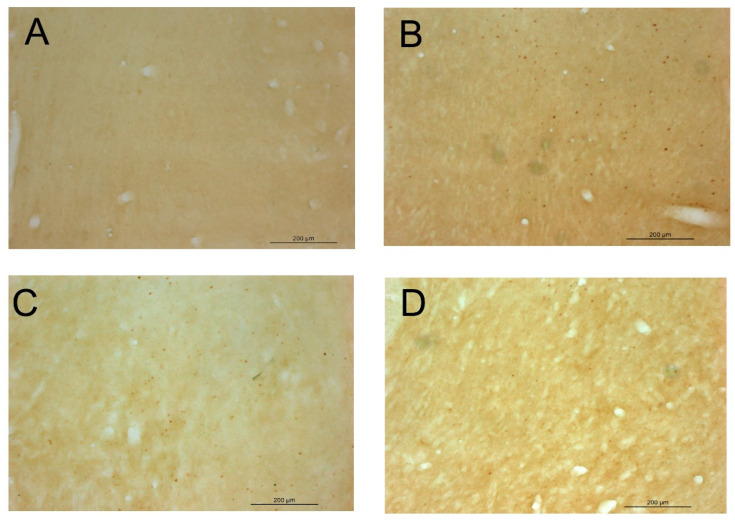
Representative images of coronal sections of the NAcc after cFOS staining in experiment 2. Legend: (**A**) group aCSF/Veh; (**B**) group aCSF/NAC 120; (**C**) group EtOH/Veh; (**D**) group EtOH/NAC 120.

As depicted in Figure 5, the direct microinjection of 150 nmol of EtOH into the VTA was able to increase the expression of cFOS IR-cells in the NAcc nearly ten times, thus demonstrating its ability to activate the mesocorticolimbic pathway and giving validity to our experimental procedure. Additionally, as observed in experiment 1, the i.p. administration of 120 mg/kg of NAC also led to a significant increase in the expression of cFOS IR-cells in the NAcc. Interestingly, the co-administration of ethanol (intra-VTA) and NAC (i.p) clearly blocked the increase in the expression of cFOS in the NAcc. Representative images of coronal sections after cFOS staining in the NAcc are available in Figure 6.

Statistical analysis confirmed the above observations. A one-way ANOVA revealed statistically significant differences between the groups (F (3, 20) = 12.36, *p* < 0.0001). On the one hand, the post hoc analysis (Tukey’s test) showed that the results obtained in the aCSF/vehicle group significantly differed from those obtained in the EtOH/vehicle group (*p* = 0.0009) as well as those obtained in the aCSF/NAC 120 group (*p* = 0.0005). On the other hand, the results obtained in the EtOH/NAC 120 group significantly differed from those obtained in the aCSF/NAC 120 group (*p* = 0.0048) and the EtOH/vehicle group (*p* = 0.0083). These results reveal that EtOH and NAC are able to significantly increase cFOS expression and further suggest that both drugs interact when simultaneously administered, such that the administration of one drug is able to counteract the effect produced by the other.

Although these preliminary results are hopeful, further experiments are needed to provide a mechanistic interpretation of this interaction. However, it could be interesting to give a possible explanation about what may have happened when NAC and ethanol were co-administered. Several studies have postulated that the NAcc responses evoked by Glu are attenuated when dopamine (DA) is present. Cepeda and colleagues reported that the responses evoked by the iontophoretic application of Glu in rat striatum slices were significantly attenuated when DA was applied [27]. More recent studies have postulated that long-term depression (LTD) of the MSNs is produced when high concentrations of DA are released in the NAcc, and that this effect is mediated by D1 receptors [28,29,30]. In addition, there is general agreement that this form of LTD is induced postsynaptically and depends upon the activation of mGluR type I [31,32,33]. Thus, one plausible explanation of the observed results under our experimental conditions could involve the interaction between Glu and DA release in the NAcc. It is well known that the intra-VTA administration of EtOH can induce a phasic DA release in the NAcc, and, as stated above, the systemic administration of a high dose of NAC can also produce an increase in extrasynaptic Glu. Therefore, the release of Glu in presence of elevated levels of DA could lead to the LTD of the the MSNs in the NAcc and, therefore, the suppression of cFOS expression in this brain area.

Our results can also be analyzed from a complementary point of view: the administration of high doses of NAC is capable of suppressing the ethanol-induced activation of the NAcc neurons. In this sense, we wonder if the blockade of cFOS expression observed in the present work could help explain, at least in part, the capacity shown by NAC to prevent the alcohol relapse that has been observed in our previous work [16]. Certainly—assuming that, in this case, the rats do not have a history of ethanol consumption—if the co-administration of a high dose of NAC and ethanol does not result in the activation of MSNs, this could help explain the suppression of ethanol relapse detected in our previous work. Obviously, further experiments are needed to corroborate this hypothesis.

## 3. Materials and Methods

### 3.1. Animals

We used 34 male Wistar rats (330–400 g) for experiment 1 and 24 male Wistar rats (300–340 g) for experiment 2. The animals were housed in plastic cages (48 × 38 × 21 cm^3^) with controlled humidity and temperature (22 °C), a 12:12 h light–dark cycle, and free access to food and water. All study procedures were carried out in strict accordance with EEC Council Directive 2010/63/UE and Spanish laws (RD 53/2013), and were approved by the Animal Care Committee of the University of Valencia and the regional government (protocol CODES: 2017/VSC/PEA/00086 approved on 29 May 2017 and 2020/VSC/PEA/0020), approved on 20 February 2020).

### 3.2. Drugs and Chemicals

The MTEP and NAC were purchased from Sigma-Aldrich Co. The MTEP was dissolved in saline, whereas the NAC was dissolved in a phosphate buffer and the pH was adjusted to 7.4. The ethanol was purchased from Scharlau (Madrid, Spain) and was freshly dissolved in an artificial cerebrospinal fluid (aCSF)/ascorbate solution prior to use. The aCSF/ascorbate solution consisted of 120.0 mM NaCl, 4.8 mM KCl, 1.2 mM KH_2_PO_4_, 1.2 mM MgSO_4_, 25.0 mM NaHCO_3_, 1.2 mM CaCl_2_, 100 mM D-glucose, and 0.2 mM ascorbate, and the pH was adjusted to 6.5.

### 3.3. Experimental Design

This study was divided into two consecutive experiments.

#### 3.3.1. Experiment 1: The Efficacy of MTEP to Suppress NAC-Induced cFOS IR-Cell Expression

In experiment 1, 34 rats were randomly assigned to 6 groups, as can be seen in Table 1:

Five days before the experimental day, rats were handled for 5 min/day, to minimize the stress provoked by the experimental procedure. The injections for treatment 1 and treatment 2 were administered consecutively. Then, 120 min after the injections, the brains were prepared as described in Section 3.7, and the cFOS IR-cells were analyzed using the immunohistochemistry techniques detailed in Section 3.8. The MTEP dose was selected according to previous research [11].

#### 3.3.2. Experiment 2: The Effect of 120 mg/kg of NAC on the Ethanol-Induced Activation of the Mesocorticolimbic System

We planned this experiment to determine whether the i.p. injection of 120 mg/kg of NAC would be able to attenuate or suppress the activation induced by the intra-VTA microinjections of ethanol. The activation of the mesocorticolimbic system was explored through the measurement of the locomotor activity displayed by the rats as well as the expression of cFOS IR-cells in the VTA-projection area (NAcc). All animals were handled for five min/day, in accordance with Section 3.6. The surgical procedure and drug administration were carried out according to Section 3.4 and Section 3.5. Two experiment subsets were arranged:

Experiment 2a—the efficacy of NAC to suppress the locomotor-activating effect of ethanol in rats. Twenty-four animals were used in this experiment and were randomly assigned to each experimental group (*n* = 6). Rats were divided into four groups depending on the intra-VTA and i.p. treatment: aCSF/saline, aCSF/NAC 120 mg/kg, 150 nmol EtOH/saline, and 150 nmol of EtOH/NAC 120 mg/kg. The ethanol dose was selected to ensure the maximum effect on locomotor activity, according to previous experiments carried out in our laboratory [18,19]. The NAC dose was selected, according to existing literature, as a NAC dose that is able to abolish the ADE effect in the EtOH relapse animal model [16]; EtOH intake and EtOH relapse, binge-like drinking [34]; and the conditioned place preference induced by EtOH [17]. The NAC administration route was selected according to previous experiments carried out in our laboratory that have used the same experimental procedure [18,19].

Experiment 2b—the efficacy of NAC to suppress the EtOH-induced expression of cFOS immunoreactive cells (IR-cells). At 120 min after ethanol/aCSF injection, the brains were removed and dissected, and the cFOS IR-cells were analyzed using the immunohistochemical technique described in Section 3.7 and Section 3.8. The study area (NAcc) was selected because it receives dense projections from VTA. In addition, NAcc and VTA are principal nuclei of the mesocorticolimbic system, a structure strongly implicated in the reward system [35].

### 3.4. Surgery and Post-Surgical Care

Surgery was performed according to [19]. The rats were anesthetized i.p. with ketamine/xylazine (i.e., 95 mg/kg of ketamine and 10 mg/kg of xylacine) and placed in a stereotaxic apparatus. An incision (8–10 mm) was made in the skin over the skull, and the wound margin was infiltrated with lidocaine (3%). Two holes were drilled: one for the skull screw and the other for the guide cannulae (Plastics One, Roanoke, VA, USA). Each animal was implanted unilaterally with a 28-gauge guide cannula aimed at 1.0 mm above the posterior VTA. The coordinates relating to the bregma and the skull surface [26] were as follows: A/P −5.8 mm; L −2.1 mm; D/V −8.1 mm. The cannulae were angled toward the midline at 10° from the vertical plane (all the measurements in the dorsal–ventral plane refer to distances along the track at 10° from the vertical plane). The cannulae assemblies were secured in place with reinforced glass cement (GC FujiCEM^®^, GC corporation, Tokio, Japan). A stainless-steel stylet (33-gauge), extending 1.0 mm beyond the tip of the guide cannula, was put in place at the time of surgery and removed at the time of testing. Following surgery, the rats were housed in individual rectangular plastic cages (42.5 × 20 × 14 cm^3^, located side by side in order to prevent the influence of chronic stress due to isolation on performance), with free access to food and water for at least 7 days. These cages were used as test cages on the day of the experiment.

### 3.5. Drug Microinjection Procedure

All of the intra-VTA drug microinjections were carried out with 33-gauge stainless steel injectors, extending 1.0 mm below the tip of the guide cannulae. Injectors were attached to a 25.0 μL Hamilton syringe using PE-10 tubing. All microinjections were carried out using a syringe pump (Kd Scientific Inc, Holliston, MA, USA), which was programmed to deliver a total volume of 200 nL in 20 s (flow rate of 0.6 μL/min). Following the infusion, the injector remained in place for 1 min to allow the diffusion of the drugs; then, it was removed, the stylet was replaced, and the locomotor activity was registered when appropriate. All the injections were administered in the experimental room.

### 3.6. Handling and Test Procedure

Two days after surgery, the animals were taken from the colony, brought to the experimental room, and handled for 5 min/day until the experimental day. During this phase, animals became accustomed to the experimenter, the experimental room, and to the injection procedure—with a total of four to seven sessions to decrease the activating effects of the manipulations taking place during the injection process, as well as the novelty-activating effects of the testing room. Tests (experiment 2) were performed 7 days after surgery. The day before the experiment, the rats were again taken from the colony room; brought to the experimental room; and placed, for 30 min, in the same type of rectangular cages in which the animals were housed. On the day of the experiment, rats were placed in their own experimental cage, and experiments started with the i.p. injection of Saline or NAC. After 30 min, ethanol or aCSF were intra-VTA injected, according to the protocol described in the drug microinjection procedures. All the experiments were recorded by a digital video camera and were analyzed for the total distance traveled (in centimeters) over 30 min, according to [19].

### 3.7. Immunochemistry

At 120 min after the drug microinjection (experiment 2) or the NAC/vehicle i.p. injections (experiment 1), the animals were deeply anesthetized with isoflurane and transcardially perfused with 200 mL of PBS, followed by 300 mL of 4% formaldehyde in PB 0.1 M. The brain was removed, and 40 mm sections were obtained, as described in [24].

Selected sections were transferred to TBS and sequentially incubated (including TBS rising between incubations) in: (1) 1% hydrogen peroxide in TBS, (2) 5% goat serum in TBS-0.3% TX, (3) an anti-cFOS polyclonal antibody (1:1000, Santa Cruz Biotechnology, Dallas, TX, USA) overnight at 4 °C, (4) a biotinylated anti-rabbit antibody (1:200; Vector Labs), and (5) an avidin-biotinylated peroxidase complex (1:200; ABC Elite Kit; Vector Laboratories, Inc., Burlingame, CA, USA). The reaction was visualized by incubating with diaminobenzidine (SigmaFAST, Sigma, St. Louis, MO, USA). Finally, the sections were mounted on slides, dehydrated in alcohols, cleared, and coverslipped for microscopical examination.

### 3.8. Image Analysis

The quantification of the cFOS IR-cells was performed in the NAcc region, selected according to [26]. Four sections per animal and area were selected, and images were digitalized using a microscope (Leica) equipped with a CCD camera. The 10× objective was selected to obtain frames of 1026 × 769 mm, and the counting of the stained nuclei per frame was carried out using the multipoint plugin of the ImageJ software (NIH, USA). The experimenter was blind to experimental grouping throughout the image acquisition and processing. Additionally, in experiment 2, sections of the VTA that were stained with cresyl violet were used for the verification of the cannulae placement.

### 3.9. Statistical Methods

Data were expressed as mean ± SD. After testing for normality with the Shapiro–Wilk test, the total distance traveled (experiment 2a) or the number of cFOS IR-cells (experiments 1 and 2b) that were determined under different experimental conditions were analyzed using a one-way ANOVA, followed by Tukey’s multiple comparison test, if necessary. Homogeneity of variance was tested before the ANOVA was performed, and the significance level was always set at *p* = 0.05. In cases where the normality test failed, a non-parametric Kruskal–Wallis test was performed. All the analyses were carried out using GraphPad Prism version 8.

## 4. Conclusions

In conclusion, the results of our experiments indicate that the higher NAC dose assayed in our experiments is able to activate glutamatergic transmission that leads to an increase in cFOS IR-cell expression in the NAcc. The negative allosteric modulator of mGLuR5 (MTEP) is able to abolish this effect, suggesting that the activating effect of NAC, when injected at a high dose, could be mediated by mGluR5. Curiously, 120 mg/kg of NAC (high dose) is able to attenuate the ethanol-induced increase in the expression of cFOS IR-cells, probably due to a phenomenon based on the LTD of the MSNs provoked by the simultaneous increase in extrasynaptic Glu and phasically released DA after the intra-VTA administration of ethanol. Further experiments are necessary to corroborate our hypothesis.

## Figures and Tables

**Table 1:** i.p. treatments in each experimental group of experiment 2.

Group	*n*	Treatment i.p. 1	Treatment i.p. 2
Veh/Sal	4	Saline	Vehicle
NAC 30/Sal	6	Saline	NAC 30 mg/kg
NAC 120/Sal	6	Saline	NAC 120 mg/kg
Veh/MTEP	6	MTEP 0.1 mg/kg	Vehicle
NAC 30/MTEP	6	MTEP 0.1 mg/kg	NAC 30 mg/kg
NAC 120/MTEP	6	MTEP 0.1 mg/kg	NAC 120 mg/kg

## Data Availability

The data reported in this study are available in this manuscript or from the corresponding author upon request.
